# Molecular Mechanisms Involved in the Multicellular Growth of Ustilaginomycetes

**DOI:** 10.3390/microorganisms8071072

**Published:** 2020-07-18

**Authors:** Domingo Martínez-Soto, Lucila Ortiz-Castellanos, Mariana Robledo-Briones, Claudia Geraldine León-Ramírez

**Affiliations:** 1 Department of Microbiology and Plant Pathology, University of California, Riverside, CA 92521, USA; 2 Tecnológico Nacional de México, Instituto Tecnológico Superior de Los Reyes, Los Reyes 60300, Mexico; 3 Departamento de Ingeniería Genética, Unidad Irapuato, Centro de Investigación y de Estudios Avanzados del Instituto Politécnico Nacional, Irapuato 36821, Mexico; lucila.ortiz@cinvestav.mx (L.O.-C.); claudia.leon@cinvestav.mx (C.G.L.-R.); 4 Departamento de Microbiología y Genética, Instituto Hispano-Luso de Investigaciones Agrarias (CIALE), Universidad de Salamanca, 37185 Salamanca, Spain; amrobledobriones@usal.es

**Keywords:** Ustilaginomycetes, smut fungi, multicellularity, filamentous growth, mycelial growth

## Abstract

Multicellularity is defined as the developmental process by which unicellular organisms became pluricellular during the evolution of complex organisms on Earth. This process requires the convergence of genetic, ecological, and environmental factors. In fungi, mycelial and pseudomycelium growth, snowflake phenotype (where daughter cells remain attached to their stem cells after mitosis), and fruiting bodies have been described as models of multicellular structures. Ustilaginomycetes are Basidiomycota fungi, many of which are pathogens of economically important plant species. These fungi usually grow unicellularly as yeasts (sporidia), but also as simple multicellular forms, such as pseudomycelium, multicellular clusters, or mycelium during plant infection and under different environmental conditions: Nitrogen starvation, nutrient starvation, acid culture media, or with fatty acids as a carbon source. Even under specific conditions, *Ustilago maydis* can form basidiocarps or fruiting bodies that are complex multicellular structures. These fungi conserve an important set of genes and molecular mechanisms involved in their multicellular growth. In this review, we will discuss in-depth the signaling pathways, epigenetic regulation, required polyamines, cell wall synthesis/degradation, polarized cell growth, and other cellular-genetic processes involved in the different types of Ustilaginomycetes multicellular growth. Finally, considering their short life cycle, easy handling in the laboratory and great morphological plasticity, Ustilaginomycetes can be considered as model organisms for studying fungal multicellularity.

## 1. Introduction

Multicellularity is considered one of the major transitions in the evolution of complex organisms on Earth. It is defined as the developmental process by which unicellular organisms became pluricellular early and repeatedly during the evolution of life, involving genetic, ecological, and environmental factors [1,2,3,4,5]. Multicellularity has different levels of complexity. In microorganisms, such as bacteria and fungi, simple multicellularity has been observed as a mechanism that increases the affinity for substrates and the obtainment of nutrients, defense against predators, tolerance to environmental stress, host colonization, and may ensure offspring [6,7,8,9,10,11,12,13]. Interestingly, two possible hypotheses have been proposed to explain the development of simple multicellularity: (i) Clonal multicellularity, where the cells are held together after mitosis and share the same genotype; and (ii) aggregative multicellularity, when unrelated cells come together to form a chimeric structure [5,14,15]. However, it has been observed that the multicellularity developed in filamentous fungi can be different from that of the previously described fungi. This is because their vegetative mycelium rarely adheres to each other except in fruiting bodies—an example of complex multicellularity, which is defined as a three-dimensional structure with a differentiated organization in time and space and a genetically determined developmental program [16,17]. Comparative genomic studies have shown that both filamentous fungi and yeasts conserve the genetic machinery for multicellular growth [16,18]. Fungal mycelium has been described as a model of a simple multicellular structure, showing a polarized syncytium that expands via tip elongation and somatic cell-cell union [19,20]. Likewise, the snowflake phenotype developed by yeasts and some fungi is considered a model of undifferentiated multicellularity [10,11,13,16].

Some fungi show multicellular growth under specific environmental conditions or development stages, e.g., *Neurospora crassa* filamentous growth, *Candida albicans* and *Yarrowia lipolytica* mycelial growth, *Saccharomyces cerevisiae* pseudomycelium and multicellular clusters, *Coprinus cinereus* fruiting bodies, *inter alia* [20,21,22,23,24,25].

Ustilaginomycetes are a group of approximately 1,185 of Basidiomycetes species, many of which are pathogens of economically important plants [26]. *Ustilago maydis* is probably the most studied species. Like other Ustilaginomycetes, *U. maydis* can grow unicellularly as saprophytic yeast-like cells (sporidia), or multicellularly as mycelium during plant infection and colonization. On the maize plant surface, sexually compatible sporidia mate to form a dikaryotic filament that penetrates the plant by forming appressorium. Mating is regulated by the *a* and *b* independent alleles. The *a* allele encodes a pheromone-receptor system and the *b* allele a *bE/bW* heterodimer transcription factor, which regulate the development of the infective dikaryotic filament [27,28,29,30,31,32]. In some of these fungi, simple multicellularity has also been observed during their growth under different environmental conditions, such as nitrogen or nutrient starvation, acid culture media, or using fatty acids as the carbon source [33,34,35,36,37]. For example, *Sporisorium reilianum* shows several types of multicellular growth: (i) As mycelium during the plant infection or under nutrient starvation conditions [29,31,33,38]; (ii) as pseudomycelium by deleting certain genes [39]; or (iii) as multicellular clusters in acidic culture medium [13]. In *U. maydis*, basidiocarp formation has also been described [40,41].

There are several mechanisms described as important for multicellular growth in Ustilaginomycetes, for example, the Mitogen-Activated Protein Kinase (MAPK) and Protein Kinase A (PKA) signaling pathways [32,42,43,44,45]; the requirement of polyamines [46,47]; epigenetic regulation [48,49,50,51]; and cell wall synthesis and degradation (a cellular structure directly involved in fungal multicellularity) [52,53,54].

Considering that the Ustilaginomycetes can grow unicellularly and multicellularly, and the importance of their morphology during development, in this review we will discuss the signaling pathways, the epigenetic regulation, the polyamine requirements, the polarized cell growth, and the synthesis or degradation of the cell wall during the multicellular growth of this class of fungi, highlighting the experimental advantages of the Ustilaginomycetes as model organisms for studying multicellularity.

## 2. Signaling Pathways Involved in the Ustilaginomycetes Multicellular Growth

**Figure 1:**
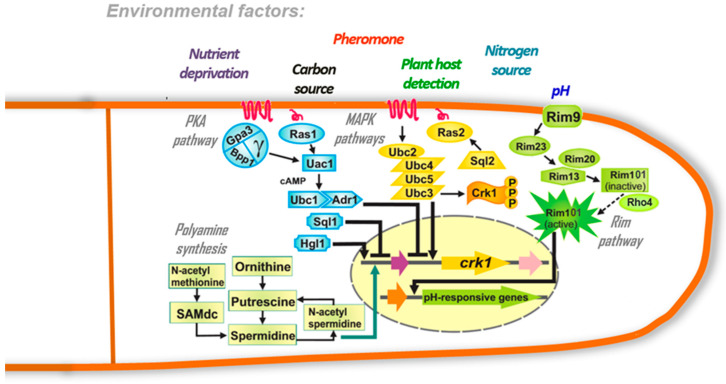
Schematic representation of the signal transduction and metabolic pathways involved in the Ustilaginomycetes multicellular growth. Several authors have suggested that the mitogen-activated protein kinase (MAPK) and cyclic AMP (cAMP)/protein kinase A (PKA) pathways are necessary for mycelium development (a simple multicellular structure observed in Ustilaginomycetes) (reviewed by References [32,45,55,56]). Polyamines, especially spermidine, are essential for the expression of the genes involved in the multicellular growth and the response to stress conditions [47,57,58,59]. Although the Rim pathway is not involved in the fungal multicellular growth, it is required to sense stressful environmental conditions such as pH [60,61,62], which is one of the most important inducers of multicellular growth in Ustilaginomycetes [13,35].

Fungi, like all living organisms, grow and develop under fluctuating environmental conditions. Therefore, they have developed mechanisms to sense, respond, and adapt to conditions that favor their growth, or to deal with adverse conditions (Figure 1).

**Figure 2:**
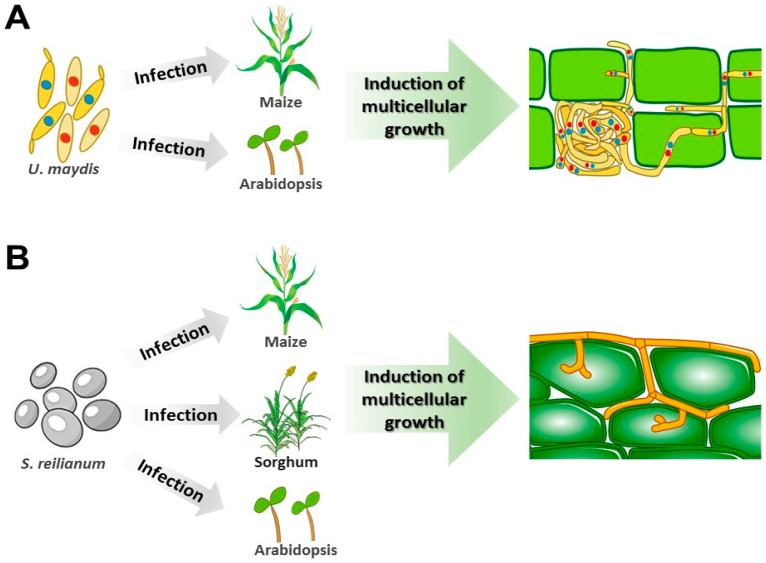
Multicellular growth of Ustilaginomycetes is induced during infection and colonization of the host plants. (**A**) Multicellular growth of *Ustilago maydis* as a filament is induced during the maize and *Arabidopsis* experimental host infection [27,63,64]. The different colors in sporidia nuclei represent different yeast-like cells that are sexually compatible. (**B**) Similarly, *Sporisorium reilianum* multicellular growth is induced during maize, sorghum, and *Arabidopsis* infection [29,31,38].

**Figure 3:**
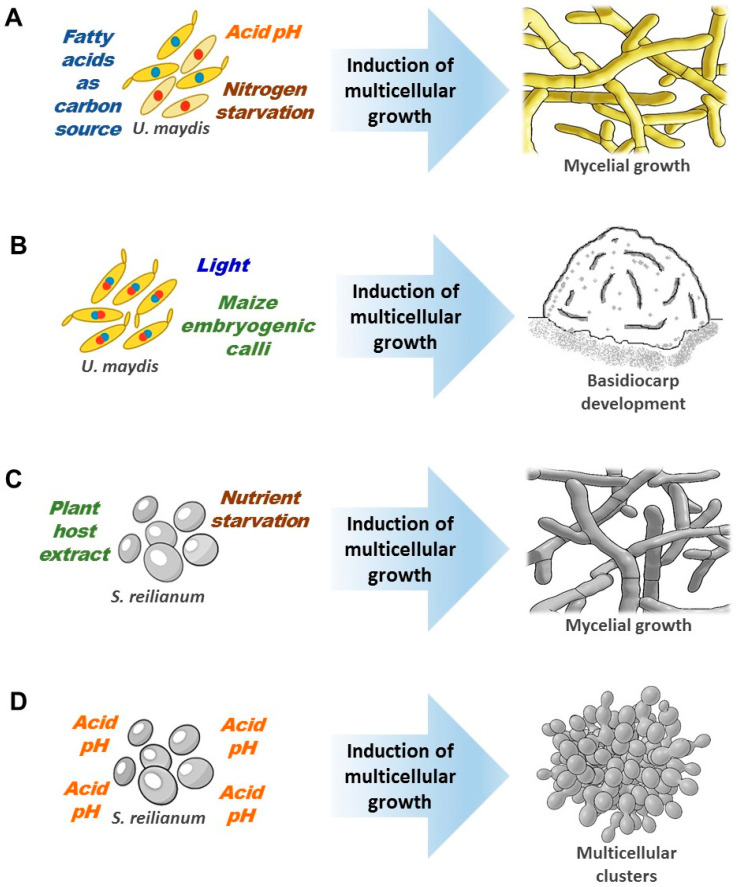
The environmental conditions induce the multicellular growth of Ustilaginomycetes. (**A**) Nitrogen starvation [34], acid culture media [35], and fatty acids as a carbon source [36] induce multicellular growth as mycelium in *Ustilago maydis*. (**B**) *U. maydis* grows multicellularly as gastroid-type basidiocarps when it is cultivated in dual cultures with maize embryogenic calli [40]. (**C**) Host plant extracts or nutrient starvation induce muticellular growth as mycelium in *S. reilianum* [33]. (**D**) Acid pH in culture medium induces multicellular cluster growth in *S. reilianum* [13].

**Figure 4:**
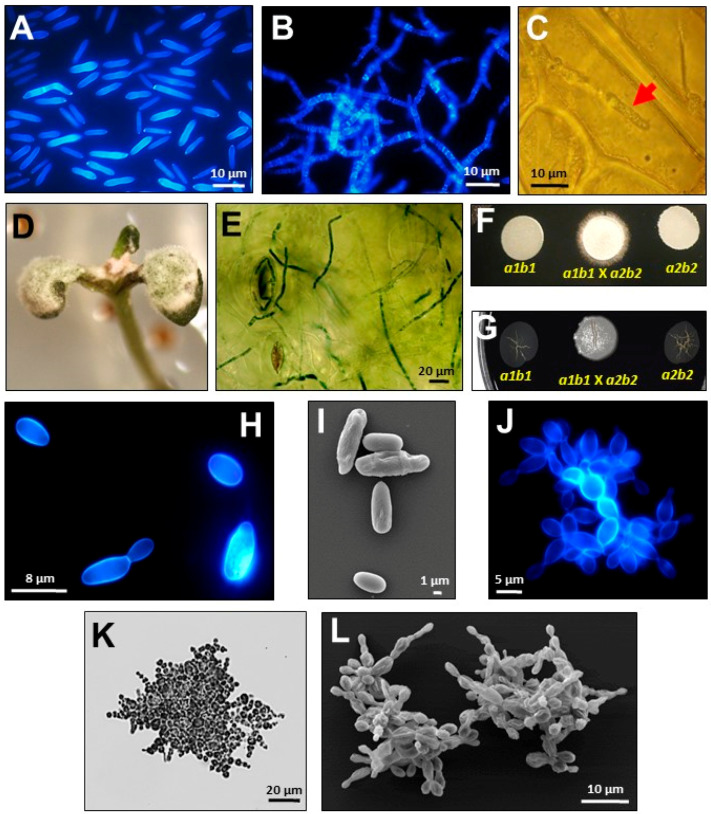
Multicellular shapes developed by Ustilaginomycetes under different growth or development conditions. (**A**–**F**), Images of *U. maydis*. (**G**–**L**) Images of *S. reilianum*. (**A**,**B**) Epifluorescence microphotographs of the fungus showing yeast-like unicellular growth, or multicellular growth as mycelium, in minimal medium pH 7 or pH 3, respectively. (**C**) Multicellular growth of the fungus as mycelium (red arrow) during the colonization of maize plant cells. (**D**,**E**) Multicellular growth of the fungus during *Arabidopsis* infection. (**F**,**G**) Multicellular growth of fungi as white-fuzzy colonies after mating of sexually compatible sporidia on plates with charcoal containing minimal medium. (**H**,**I**) Epifluorescence and scanning electron microphotographs of the fungus growing unicellularly like yeasts. (**J**–**L**) Epifluorescence, bright field, and scanning electron microphotographs of the multicellular clusters of the fungus. In all the epifluorescence microphotographs, the fungi were stained with calcofluor white (Sigma-aldrich, 18909). In (**E**,**K**), the fungi were stained with cotton blue-lactophenol (Sigma-Aldrich, 61335, St. Louis, MO, USA).

Multicellular growth is induced in Ustilaginomycetes: During plant infection and colonization, due to nitrogen or nutrient starvation, in acidic culture media and using fatty acids as carbon sources. *U. maydis*, for example, can grow multicellularly as basidiocarp in the presence of maize embryogenic calli (Figure 2, Figure 3, and Figure 4B–G,J–L) [13,29,32,33,34,35,36,40].

Although pH is one of the environmental factors that induce multicellular growth in some Ustilaginomycetes (Figure 3A,D and Figure 4B,J–L), the Pal/Rim pathway, the main mechanism fungi use to sense pH (Figure 1) [61,65], is not involved in *U. maydis* multicellular growth [60,61,62], and may not be involved in other Ustilaginomycetes growth either (the Pal/Rim pathway is conserved in smut fungi, see Table 1). We will discuss the MAPK and PKA signal transduction pathways involved in Ustilaginomycetes multicellular growth in response to the different conditions or factors previously mentioned. 

### 2.1. MAPK Pathways

In eukaryotic organisms, including Ustilaginomycetes, the MAPK signaling pathway is one of the most conserved and important mechanisms for the transduction of information from the exterior to the interior of the cell (Table 1). In the MAPK pathway, stimuli are perceived by receptors and proteins anchored in the cell membrane. The receptors can be G-protein-coupled to transmembrane receptors, receptor tyrosine kinases (RTKs) or two-component signal transduction systems (TCS). When the receptors perceive the stimuli, a sequential activation is induced by phosphorylation of serine/threonine residues in the three protein kinases: MAP kinase kinase kinase (MAPKKK), MAP kinase kinase (MAPKK), and MAP kinase (MAPK) (Figure 1). The MAP kinase activates the transcription factors that translocate to the nucleus to regulate the expression of the genes in response to the external stimuli [45,72,73].

In fungi, the MAPK signaling pathway regulates physiological processes, such as the cell cycle, multicellularity, sporulation, mating, cell wall synthesis and integrity, response to stress, and virulence [45,74,75]. The *Saccharomyces cerevisiae* and *Candida albicans* MAPK signaling pathways have been extensively studied, so there is more information available. The *S. cerevisiae* Kss1 MAPK signaling pathway is necessary for multicellular pseudomycelium growth. The *C. albicans* homologous pathway is known as Cek1, and *U. maydis* homologous pathway is known as PMM (pathogenesis, mating, multicellularity, or morphogenesis). In *U. maydis*, the MAPK PMM pathway controls the multicellular growth in vitro [induced by different stress conditions (Figure 3A,B and Figure 4B)], and in vivo [during host plant infection, (Figure 2A and Figure 4C–E)] [32,45]. The deletion of *C. albicans* and *U. maydis* MAPK pathway components suppresses the multicellular formation of pseudomycelium and mycelium, respectively [22,32,44,76,77,78,79,80].

In *U. maydis*, the proteins of the MAPK core are known as: Ubc4/Kpp4 MAPKKK, Fuz7/Ubc5 MAPKK and Ubc3/Kpp2 MAPK (Figure 1). They are important for mating, pathogenicity, and the formation of multicellular mycelium. They show homology with the protein kinases *STE11*, *STE7* and *KSS1* of the *Saccharomyces* Kss1 MAPK pathway [81,82,83,84,85,86].

In the *Ustilago esculenta* and *Sporisorium scitamineum* Ustilaginomycetes, the protein kinase Kpp2 has the same function as its ortholog in *U. maydis* [87,88]. Phylogenetic analysis showed that the Kpp2 sequence is highly conserved among smut fungi [87]. In *U. esculenta*, the *UeKPP2* gene is up-regulated in multicellular filamentous growth, and UeKpp2 interacts with Uefuz7 and UePrf1, the same as in *U. maydis* [88]. Interestingly, Δ*Sskpp2* showed a decrease in mating and filamentation, which was partially restored by adding tryptophol, a quorum-sensing (QS) aromatic alcohol secreted by yeasts that stimulates *S. cerevisiae* and *C. albicans* mycelium or pseudomycelium growth, respectively. In *S. cerevisiae*, production of these aromatic alcohols is regulated by extracellular nitrogen and cell density. For example, high concentrations of nitrogen sources repress its synthesis, while low concentrations activate it. However, no multicellularity regulation by QS has been observed in *U. maydis* or other Ustilaginomycetes [87,89,90].

In Ustilaginomycetes, upstream of the MAPKs PMM core is the Ubc2 adaptor protein (Figure 1), an orthologue of Ste50 from *S. cerevisiae*. Ubc2 contains the domains: SAM (sterile α motify), RA (Ras association), and in two SH3 C-terminals (Src homology 3). All of them are important for protein-protein interaction. For example, through the SAM domain, Ubc2 interacts with ubc4/kpp4 (MAPKKK) protein kinase [79,91]. *UBC2* is necessary for *U. maydis* multicellular mycelial growth. The ∆*ubc2* strains that were affected in the MAPK PMM pathway showed a severe reduction in the formation of dikaryotic filamentous and haploid mycelium, which was induced under in vitro conditions [44,91]. Interestingly, the transcriptomic analysis of Δ*ubc2* showed that 939 genes [≈ 14.0 % of the *U. maydis* genome (6883 genes)] are directly or indirectly regulated by the MAPK PMM pathway [80]. Among the genes natively regulated by the inactivation of MAPK PMM, there were genes encoding proteins involved in membrane synthesis or cell wall synthesis [chitin synthases, chitin deacetylases, Kre6 (glucan synthase), Rot1 (involved in cell wall function), chitin-binding proteins, exo-1,3-beta-glucanase precursors]; genes encoding proteins involved in multicellular growth (actin cytoskeleton organization, myosins, kinesins); genes encoding protein kinases and serine/threonine kinases, with *CRK1* between them; and genes encoding the GTPases, such as *RAS* and *SQL2* [80].

The Ras proteins are small monomeric GTPases that act as molecular switches in the signaling pathways that alternate between the GTP and GDP-bound forms in response to extracellular stimuli. In *U. maydis*, the *SQL2* gene encoding a CDC25-like protein is involved in the activation of Ras2 [92]. The constitutive activation of *RAS2* or the overexpression of *SQL2* promotes the filamentous multicellular growth of this fungus, even in haploid strains [92,93]. *S. cerevisiae* Ras2 acts in the same way in the MAPK pathway during pseudomycelial growth [94].

The MAPK pathway is conserved in Ustilaginomycetes (Table 1). As occurs in *U. maydis*, the MAPK PMM pathway may interact antagonistically with the cAMP/PKA pathway to regulate unicellular (Figure 4A,H,I) or multicellular growth (Figure 4B–G,J–L) [32,45,95] in other Ustilaginomycetes. MAPK PMM is required for multicellular growth as mycelium, and PKA for unicellular growth as yeast. The mutant strains in the MAPK cascade genes, e.g., *UBC2*, *UBC3*, *UBC4*, and *UBC5*, showed a constitutive yeast growth [81,83,84,85,91]. The PKA and MAPK signal transduction pathways converge with Crk1, a Ser/Thr kinase protein. The Δ*crk1* mutant strains, unlike the wild type strains, grow constitutively as unicellular yeasts on acidic culture medium and under nutrient starvation conditions [95]. However, the *CRK1* gene is also necessary for the filamentous growth induced by defects in the cAMP/PKA pathway [95]. Likewise, in *S. scitamineum*, the MAPK and PKA pathways show an antagonistic interaction during multicellular or unicellular growth regulation. The Δ*Sskpp2* mutant strains show a reduction in filamentation, which is partially restored by adding cAMP [87].

As mentioned above, *U. maydis* can form large hemi-spheroidal structures with gastroid-type basidiocarp characteristics (a complex multicellular structure) when grown in solid medium supplemented with auxins in dual cultures with maize embryogenic calli (Figure 3B) [40]. In this fungus, the MAPK PMM pathway is involved in the formation of basidiocarps. The Δ*Fuz7* mutant strains did not develop basidiocaps, and several genes encoding serine/threonine kinases were up-regulated during the early stages of basidiocarp formation, e.g., Kpp2 and Kpp6 were 2.2 and 11.4 times overexpressed, respectively. Similarly, this occurred during the development of *Coprinopsis cinérea* fruiting body, when the MAPK pathway genes are up-regulated [96]. Finally, during the development of multicellular clusters of *Sporisorium reilianum* (Figure 3D and Figure 4J–L), the important role of several MAPK proteins and serine/threonine kinases was suggested by transcriptional network analyses [13].

### 2.2. cAMP/PKA Pathway

In eukaryotic cells, the secondary messenger, cyclic adenosine monophosphate (cAMP) is produced in response to extracellular stimuli. When the receptors perceive a stimulus, a dissociation of the α-subunit of the G-protein is induced in order to activate or repress the cAMP synthesis by the adenylate cyclase enzyme (Uac) (Figure 1). When the levels of cAMP are low, the PKA is an inactive tetramer compound of two catalytic and two regulatory subunits. However, when the levels are high, cAMP binds to a regulatory subunit causing a holoenzyme dissociation in a dimeric regulatory subunit and two active monomeric catalytic subunits which phosphorylate transcription factors and metabolic enzymes [97,98,99].

In fungi, the cAMP/PKA signaling pathway has a function in mating, sporulation, dimorphism, response to stress, and virulence [77,100,101]. In *S. cerevisiae* and *C. albicans*, both cAMP/PKA and MAPK pathways participate in the shift from unicellular growth as yeast to multicellular growth in the form pseudomycelium or mycelium, respectively [22,77,78,102]. In contrast, and as described above, cAMP/PKA and MAPK PMM are antagonistic in *U. maydis* [32,45,55]. In this fungus, the inactivation of the cAMP/PKA pathway by deleting the genes that encode adenylate cyclase (Uac1) or a regulatory subunit of PKA (Adr1) showed a constitutive phenotype of multicellular haploid mycelium. In addition, the unicellular budding growth was restored when exogenous cAMP was added to the culture medium [42,43]. The same phenotype was observed in Δ*gpa3* and Δ*bpp1* mutants, that were affected in the α- and β- heterotrimeric GTPase subunits, respectively. The cAMP/PKA pathway in *Ustilago hordei* acts in the same way as in *U. maydis*. The deletion in the heterotrimeric GTPase α-subunit (Δ*fil1*) produced constitutive mycelial growth in a solid and liquid medium, and the addition of exogenous cAMP restored the budding growth [103].

Although the cAMP/PKA signaling pathway is highly conserved in Ustilaginomycetes (Table 1), in *S. scitamineum*, and contrary to *U. maydis* and *U. hordei*, the cAMP/PKA pathway is involved in multicellular growth as mycelium. The deletion of different pathway components (Δ*ssgpa3*, Δ*ssuac1* and Δ*ssadr1*) suppresses the formation of dikaryotic mycelium. The production of cAMP in Δ*ssauc1* was blocked, and severely reduced in *∆ssgpa3* and Δ*ssadr1*. The mycelial growth was restored in Δ*ssgpa3* and Δ*ssuac1* by adding exogenous cAMP, but not in Δ*ssadr1*. This makes sense because SsGpa3 and SsUac1 act upstream of cAMP production [104]. Moreover, in this smut fungus, the AGC protein kinases (SsAgc1) regulate the signaling pathway involved in mating and dikaryotic filamentous growth; therefore, this protein is crucial for the expression of genes involved in multicellular growth of *S. scitamineum* [105].

Other cAMP/PKA pathway components and their functions during *U. maydis* unicellular-multicellular growth have been analyzed. An example of this is the *HGL1* gene (hyphal growth locus), which encodes a regulator of the unicellular and multicellular morphologies and is important for mating and teliospore formation during the fungus’ pathogenic process [106]. Moreover, the *SQL1* gene encoding a repressor of the *U. maydis* cAMP/PKA pathway is itself a repressor of unicellular fungus growth [107].

In the early developmental stages of *U. maydis* basidiocarps (a complex multicellular structure), the gene encoding the cAMP-dependent protein kinase catalytic subunit was 2.3 times overexpressed [41]. In this regard, in *C. cinerea*, *Schizophyllum commune* and *Volvariella volvacea*, the cAMP content is essential for the formation of the fruiting body [108,109,110,111], and the genes of cAMP/PKA signaling pathways are up-regulated during the development of the *C. cinerea* fruiting body [96]. Finally, the participation of the kinase A protein and cAMP-dependent protein kinase in the formation of *S. reilianum* multicellular clusters was predicted by transcriptional network analyses [13].

## 3. The Role of Polyamines in the Multicellular Growth of Ustilaginomycetes

Polyamines are aliphatic polycations that are present in all living organisms. They are essential for development and growth [112]. The polyamines modulate the enzymatic activities, gene expression, DNA-protein interactions, and protect DNA from enzymic degradation [113,114]. In fungi, they regulate spore germination, appressorium formation, conidiation and multicellular growth in the form of mycelium [46,57,115,116,117,118,119]. The common polyamines in some fungi are putrescine, spermidine, and spermine. However, Ustilaginomycetes, such as *U. maydis* do not have spermine [59,120,121], and they conserve the same genes for polyamine synthesis (Table 2, Figure 1). This characteristic, and the fact that these fungi can grow as unicellular and multicellular forms, make them excellent model organisms for studying the metabolism and function of polyamines in fungi multicellularity.

Strains of *U. maydis* with *ODC* gene mutated, which encode ornithine decarboxylase (Odc) and is responsible for putrescine synthesis, are unable to grow as mycelia under acidic pH conditions with limiting concentrations of putrescine [46]. In basidiomycota fungi, such as smut fungi, the spermidine synthase (Spe) is encoded by a chimeric bifunctional gene that also encodes saccharopine dehydrogenase (Sdh), an enzyme involved in lysine synthesis [121]. The *U. maydis* Δ*spe-*Δ*sdh* mutant strains are auxotrophic for lysine and spermidine and require high concentrations of spermidine in order to grow multicellularly as mycelium under acidic conditions in culture medium [57]. Interestingly in Ustilaginomycetes, there is an alternative mechanism for putrescine synthesis, through the polyamine oxidase enzyme involved in the retroconversion of spermidine to putrescine. *U. maydis* double mutants (Δ*odc*/Δ*pao*) in the genes encoding Odc and Pao grow multicellularly as mycelium with the addition of spermidine only and in complete absence of putrescine [58]. This indicates that spermidine is the most important polyamine for multicellularity in Ustilaginomycetes [58,59,119,122].

## 4. Epigenetic Regulation of Multicellular Growth in Ustilaginomycetes

In fungi, as in all eukaryotic organisms, DNA is wrapped and highly compacted around histone proteins forming the nucleosomes within the nucleus. This organization can be altered by post-translational modifications of histones that relax or compact the nucleosomes: Acetylation by histone acetyltransferases (HATs), or deacetylation by histone deacetylases (HDACs), respectively. Nucleosome relaxation allows accessibility of DNA to different proteins involved in replication and gene transcription in response to environmental signals.

Although Ustilaginomycetes have homologous genes encoding HATs and HDACs (Table 3), the epigenetic regulation of multicellular growth has only been studied in *U. maydis* [48,49,50]. In this fungus, the *GCN5* HAT was deleted. The mutant strains were slightly more sensitive to different stress conditions than the wild type, but they grew multicellularly as mycelium and fuzz-like colonies (constitutive mycelial growth) under all the growth conditions analyzed. Also, the virulence was dramatically reduced [48]. Through transcriptomic analysis of mutant and wild type strains, it was observed that in the mutant strain (Δ*gcn5*) a total of 1203 were differentially regulated. Of these, 574 were repressed and 629 were overexpressed. Interestingy, 67 genes described as important for yeast growth (unicellularity, Figure 4A) and 66 described as important for mycelial growth (multicellularity, Figure 4B) [123], were down-regulated and up-regulated, respectively in the Δ*gcn5* mutant [49]. Among the differentially expressed genes, the following phenomena stand out: Genes involved in cell wall biogenesis and genes related with pathogenesis were down-regulated; genes required for mycelium development were overexpressed. For example, the *REP1* gene that encodes a repellent protein was 33.2 times up-regulated in the mutant strain [49].

Contrary to the deletion of *GCN5* HAT, the deletion of *HOS2* and *CLR3* HDACs affected *U. maydis* multicellular growth. In the Δ*hos2* mutant, the formation of the conjugation tube and the mating of compatible single-cell strains were altered. In addition, the multicellular dikaryotic mycelium and virulence were reduced [50].

In summary, the HATs and HDACs play an indispensable, but opposed, role in *U. maydis* multicellular growth and possibly in other Ustilaginomycetes fungi. While the histone acetylation by Gcn5 allows the expression of genes involved in unicellular growth as yeast (Figure 4A), histone deacetylation by Hos2 and Clr3 allows multicellular growth as mycelium (Figure 4B). Histone deacetylation has been usually associated with gene repression. However, these findings demonstrate that the HDACs also function as transcriptional activators [51]. Considering that these genes are evolutionarily conserved in eukaryotic organisms such as Ustilaginomycetes (Table 3) [124,125,126,127,128,129], and that most eukaryotic organisms are multicellular, it is tempting to speculate about the importance of this histone acetylation or deacetylation switch during the developmental process by which unicellular organisms became multicellular during evolution.

Another epigenetic mechanism suggested in the regulation of the multicellular growth of Ustilaginomycetes is DNA methylation. Different DNA methylation patterns were observed during the unicellular and multicellular growth of *U. maydis* wild-type strains [130]. Interestingly the mutants affected in the putrescine synthesis (Δ*odc*), which could not grow multicellularly as mycelia (discussed above) [46], showed DNA methylation patterns similar to those of the wild-type yeast [131]. Knowing that polyamines bind to DNA [132], and that DNA methylation is involved in gene silencing [133], it suggests that polyamines, particularly spermidine, prevent DNA methylation, thus allowing the expression of genes involved in *U. maydis* multicellular growth [134]. Taking into consideration that the genes involved in the polyamine synthesis are conserved in Ustilaginomycetes (Table 2), it is possible that this regulatory mechanism of multicellular growth is present in this class of fungi.

## 5. The Cell Wall in the Multicellular Growth of Ustilaginomycetes

The cell wall is the rigid outer structure that covers the cells of prokaryotic and eukaryotic organisms. It protects the cells from osmotic pressure differences between the cytoplasm and the external medium and gives shape to the cell. In fungi, the cell wall is a compound of polysaccharide microfibrils (chitin and β-1,3-glucans) immersed in a glycoprotein matrix [135]. This cellular organelle is a dynamic structure whose composition changes during the cell cycle, depending on the environmental conditions. It is also directly involved in the unicellular or multicellular development of fungi. The cell wall components are synthesized in defined quantities, according to time and space to form a coherent and organized structure [136,137]. 

Chitin is the characteristic component of the fungal cell wall, giving it rigidity. It is synthetized by the chitin synthase proteins (Chs), which are encoded by the genes known as *CHS*. Fungi have multiple *CHS* genes in their genomes, and it can be a compensatory mechanism of the cell in response to the loss of a certain proportion of them. *S. cerevisie* has three *CHS* genes: *CHS1*, which has a repair function during cell separation, allowing unicellular growth [138]; *CHS2* is involved in cell division, and it is essential for septum formation during multicellular growth [139]; and *CHS3*, which is responsible for chitin synthesis in the ring at bud emergence, for the chitin synthesized in the cell wall, and for chitosan synthesis in ascospores [140]. In multicellular fungi, such as *N. crassa* and *Aspergillus nidulans*, the *CHS* genes are necessary for normal hyphae development [141,142].

Ustilaginomycetes have approximately eight *CHS* genes (Table 4). In *U. maydis*, the Δ*chs6* mutants showed morphological alterations that were more notorious during the multicellular growth as mycelium [143]. Chs6, Chs5, Chs7 and Chs8 are localized in the growth zone in the hyphal tip, which indicates that these could contribute to polarized growth during multicellularity in the smut fungus [144]. Interestingly, the *CHS* genes are differentially expressed during the dimorphic transition of several fungi, e.g., *Mucor circinelloides* and *Paracoccidiodes brasiliensis*, showing high levels of expression in the mycelium form [145,146]. In *U. maydis*, its eight *CHS* genes were expressed in both yeast (unicellular growth, Figure 4A) and mycelium forms (multicellular growth, Figure 4B), and similarly, as in the previously described fungi, almost all the *CHS* genes showed high levels of expression in the mycelium [52].

Chitosan is another component of the fungal cell wall, although its function is less understood than that of chitin. Chitosan is synthetized through the partial deacetylation of chitin by chitin deacetylases (Cda). *S. cerevisiae* has two *CDA* genes, *CDA1* and *CDA2*, that are expressed only during sporulation. *CDA1* and *CDA2* have redundant functions in chitosan synthesis in ascospores, which contributes to their resistance to lytic enzymes [147]. Of the ten *Magnaporthe oryzae CDA* genes, only *CDA1* showed high levels of expression during its multicellular filamentous growth. However, Δ*cda1* mutat did not show alteration in the chitin content of the wall. Moreover, Cda1 was localized in the cell wall and septa during hyphae multicellular growth, but not in the growth zones. These findings suggest that *CDA* has an indirect function in hyphae morphology [148]. Ustilaginomycetes have several *CDA* genes; for example, *U. maydis* has eight *CDA* genes (Table 4). *CDA1* was 40 times up-regulated in the multicellular mycelial form [53], and the Δ*cda1* mutants developed aberrant hyphae [149]. 

Polysaccharide β-1,3-glucans are the major components of the cell wall, which are synthetized by the β-1,3-glucan synthases (Gls). In *S. cerevisiae*, the genes that encode the catalytic subunit of Gls enzymes are known as *FKS*, due to their sensitivity to FK506 immunosuppressants, but in other fungi, they are known as *GLS*. *N. crassa* has one *GLS-1* gene. gls-1-RNAi transformants show a decrease in the β-1,3-glucans synthesis, and abnormalities in the development of the multicellular mycelium; the hyphae were shorter than those of the wild type strain [150]. Gls-1-GFP accumulates at the apex of the hyphae forming a ring structure in the outer region of the Spitzenkörper [151]. In the Ustilaginomycete *U. maydis*, the *GLS* gene showed constitutive expression during multicellular growth like mycelium [52].

On the other hand, in the different stages of fungi development and multicellular growth, the cell wall is remodeled through the synthesis and degradation of their compounds. The cell wall degradation by chitinases and glucanases causes plasticity, which allows the introduction of new synthetized components by forming links between polymers and leading to the expansion of the cellular surface through turgor force [136,137].

The chitinases (Cts) remodel chitin in the cell wall. In *S. cerevisiae*, the endo chitinase Cts1 is necessary for unicellular growth, allowing cellular separation after cytokinesis. The Δ*cts1* mutants showed abnormal separation during budding growth, forming cellular clusters [152]. During budding (unicellular growth), Cts1 is localized at the mother-daughter neck, contributing to the degradation of the septum, and allowing cell separation [153]. In *C. albicans*, deletion of the *CTS1* and *CTS2* chitinase genes increases the development of pseudomycelium or cellular clusters [154]. Interestingly, the *N. crassa CTS-1* gene is also important for the development of mycelium, which suggests that it has a function in remodeling the cell wall during fungus multicellular growth. The Δ*cht-1* mutants showed reduced mycelial growth compared to that of the wild- type strains [155]. In Ustilaginomycetes, there are approximately five putative chitinase genes (Table 4). However, they may have redundant functions. In *U. maydis*, only the double mutant Δ*cts1/*Δ*cts2*, but not the single mutants, showed alterations in cell separation. Cts1 and Cts2 are active during unicellular growth as yeast, but only Cts1 showed activity during multicellular growth as mycelium [54]. However, *CTS2* was 6.7 times up-regulated in the *U. maydis* mycelium induced by the acidic pH in the culture medium. [53,123]. Likewise, the β-1,3-endo and exo-glucanases have a function in septum separation, and the β-1,3-glucans in elongation and reorganization. *ENG1* encodes a β-1,3-endoglucanase in *S. cerevisiae*. The deletion of *ENG1* shows alteration of the separation of septae and induces growth of mutants as multicellular clusters. Eng1 is mainly localized at the bud neck on the daughter side [156]. Ustilaginomycetes have several putative glucanases (Table 4). However, little is known about their function during unicellular or multicellular growth in these fungi.

Finally, the putative importance of genes involved in cell wall synthesis and structure during development of *S. reilianum* multicellular clusters was determined by transcriptional network analysis [13]. The examples of genes involved include those encoding: 1,3-beta-D-glucan synthase, glucan 1,3-beta-glucosidase, GPI-proteins, UTR2-cell wall proteins, chitin synthase 2 (Chs2), Chs3, exo-1,3-beta-glucanase, Kre6-glucan synthase, chitin deacetylases (Cda), glucan 1,3-beta-glucosidase, and several proteins involved in cell wall biogenesis and architecture. Some of them, such as *UTR2*-cell wall, *CHS3*, *ECM4*, involved in cell wall biogenesis and architecture, and *CDA*, were down regulated in the multicellular cluster form of the fungus [13]. All these findings demonstrate the direct and critical role of the cell wall in fungi multicellularity. It is tempting to hypothesize that the cell wall is the most important structure involved in this phenomenon in fungi.

## 6. Polarized Growth during the Multicellularity of Ustilaginomycetes

Mycelia or filamentous growth is one of the best-known forms of simple multicellular structures of fungi. The mycelia are joined and elongated cells in the form of tubes, which extend at the tip and branch out into sub-apical areas involving a coordinated action of the microtubule and actin cytoskeletons [157]. In Ustilaginomycetes, this phenomenon has been studied most in *Ustilago maydis*, although the genes involved in polarized growth are mostly conserved in this class of fungi (Table 3).

The actin cytoskeleton in *U. maydis* is formed by three structures: Actin cables, actin patches and an actin ring. During the multicellular growth, the hyphae contain an actin cap at the tip, and the actin cables polarize towards the tip [158]. *LIS1* is a gene encoding a microtubule plus-end tracking protein required for microtubule cytoskeleton organization, cell wall integrity, septa positioning, and nuclear migration during the filamentous growth. The Δ*lis1* mutants showed an aberrant multicellular morphology [159]. Likewise, the *TEA4* gene is involved in the polarized growth, the control of position and the number of septa, as well as the nuclear division in the mycelial form [160]. Also, *FUZ1*, a gene encoding the MYND Zn finger domain protein, is important for the cell wall integrity and dikaryotic filamentous growth after the fusion of compatible sporidia [161].

In Ustilaginomycetes, as in all the filamentous fungi, the kinesin proteins are involved in vesicle transport. *U. maydis* has ten kinesins: Kin1, Kin3, Kin4, Kin6, Kin7a, Kin7b, Kin8, Kin9, and Kin14. Of all these proteins, only Kin1 and Kin3 are important for normal multicellular filamentous growth. Δ*kin1*, Δ*kin3* and the double mutants Δ*kin1/*Δ*kin3* show bipolar growth and short hyphae [162]. Kin1 and Kin3, as well as the myosin Myo5 and Tea1, localize and accumulate at the hyphal apex during multicellular growth [162,163,164], although Tea1 is also localized in new sites of the filamentous growth [164]. The mutation of the *MYO5* gene (Δ*myo5*) did not affect the filamentous growth of the fungus. However, it caused irregular growth and irregular chitin deposition [163], thus demonstrating the important relationship between the fungi multicellular filamentous growth and their cell wall.

## 7. Other Cellular Processes Involved in the Ustilaginomycetes Multicellular Growth

Ustilaginomycetes are model organisms to study the different processes that occur in fungi. Several genes involved in the cellular processes associated with their multicellular growth have been described. Interestingly, these genes are conserved in this class of fungi (Table 3), and it is possible that those genes and the cellular processes work in the same way in Ustilaginomycetes.

In *U. maydis*, it has been observed that *REP1*, *HUM2*, and the *HUM3* genes encode repellent proteins and hydrophobins, a group of small cysteine rich proteins required for the development of multicellular aerial hyphae. The Δ*rep1* and Δ*hum2* mutants showed a drastic reduction of aerial mycelium [165,166]. Moreover, *HUM2* and *HUM3* were overexpressed 9.1 and 14.7 times, respectively, during the formation of the hyphal layer in *U. maydis* basidiocarps [41].

Likewise, the importance of the *PEP4* gene encoding the vacuolar acid proteinase PrA during *U. maydis* mycelial growth under stress conditions and pathogenesis has been reported. The Δ*pep4* mutants showed a severe reduction of multicellular mycelium growth induced by the acidic culture medium or using fatty acids as a carbon source [167]. The GTPases Cdc4 and Rho1 also played essential roles during the polarized growth and filamentation of this fungus [168,169]. In addition, the RNA-binding protein Rrm4 was crucial for the mycelium polarity during infection, because it is involved in the microtubule-dependent transport of mRNAs during fungus multicellular growth [170].

In *S. cerevisiae* and other fungi, a low level of nitrogen in the medium induces multicellular growth of pseudomycelium or mycelium [72]. An important mechanism that links the availability of nitrogen and the unicellular-multicellular growth is are the methyl ammonium permeases (Mep). In *U. maydis*, the *MEP1* and *MEP2* genes, are important for the filamentous development in a medium with low ammonium. The Δ*ump2* mutants did not grow as mycelia, and the Δ*ump1/*Δ*ump2* double mutant showed multicellular aggregates and sediment in liquid media with low ammonia concentrations. Moreover, it was suggested that Mep2 is phosphorylated by the cAMP/PKA signaling pathway in response to low nitrogen conditions [171]. Interestingly, the genes encoding the protein involved in the export of ammonia were up-regulated during *U. maydis* multicellular growth induced under acidic conditions [123]. Similarly, the *SSA2* ammonium permease was up-regulated in *Sporisorium scitamineum* during unicellular growth induced by mycophenolic acid, which is a fungal mycelium inhibitor [172]. Also, in *S. scitamineum*, the deletion of the *SSATG8* gene encoding the Atg8 protein involved in autophagy caused multicellular growth as pseudomycelium and hypersensitivity to oxidative stress [173].

Finally, in the Ustilaginomycete *Ustilago esculenta*, a pathogenic fungus of *Zizania latifolia*, the addition of exogenous arginine or the deletion of the UeArginase gene inhibited its multicellular growth as mycelium and reduced the expression of the *UeKPP6*, *UePKAC* and *UePRF1* genes, thus suggesting that arginine acts as an inhibitor of the cAMP-PKA signaling pathway [174].

All these findings demonstrate that multicellular development in Ustilaginomycetes is a holistic phenomenon, and that it is necessary to deal with the whole cell to the largest possible extent.

## 8. Conclusions

Multicellularity is defined as the developmental process by which unicellular organisms have become pluricellular during evolution. The Ustilaginomycetes are eukaryotic microorganisms with the capacity to grow unicellularly in the form of saprophytic yeasts or like undifferentiated and simple multicellular structures: Mycelium, pseudomycelium, and cellular clusters. Complex multicellular structures have also been described in the case of *U. maydis* through the formation of basidiocarps.

The data discussed in this study show that the molecular mechanisms involved in multicellular growth may be conserved in Ustilaginomycetes. These fungi have an important set of genes and regulatory mechanisms involved in their unicellular and multicellular growth. Examples of these mechanisms are: The MAPK and cAMP/PKA signaling transduction pathways, the mechanisms of epigenetic regulation, the metabolic requirements, the polarized cell growth, and the processes of cell wall synthesis and degradation. However, although these fungi are phylogenetically related, they can respond with different morphologies to. For example, *U. maydis* grows as mycelium in acidic culture media, while *S. reilianum* grows in the form of multicellular clusters under the same conditions.

Basically, the MAPK and PKA pathways transduce environmental signals to the cell nucleus by the activation of transcription factors, which bind to DNA for the transcription of target genes involved in the Ustilaginomycetes unicellular or multicellular growth. Putatively, the transcription of target genes requires the binding of polyamines (spermidine) to DNA, avoiding its methylation and allowing its transcription. Undoubtedly, the coordinated acetylation or deacetylation of histones is required to access the target DNA and its transcription.

As described above, Ustilaginomycetes multicellularity can be induced in vitro by different kinds of stress or induced in vivo as a fundamental requirement for host plant infection and colonization. Regarding host plant infection, it is well known that mating of compatible sporidia induces filamentous growth in the fungus. In addition, it is well established that many phytopathogenic Ustilaginomycetes have a limited range of plant hosts. However, little is known about the plant compounds that induce the multicellular filamentous development for host plant colonization; or about the plant compounds sensed by Ustilaginomycetes to recognize potential hosts. It would also be important to increase our knowledge of how cell-cell communication occurs in the different multicellular forms developed by the Ustilaginomycetes. It is possible that the communication between cells is also carried out through the MAPK and cAMP/PKA signaling pathways.

There are many cellular processes involved in fungi multicellularity, and it is important to understand that multicellularity is a holistic phenomenon. Therefore, it is necessary to deal with the whole cell. Finally, taking into consideration the morphological plasticity presented by Ustilaginomycetes, the availability of their sequenced genomes, their short life cycle, and easy handling in the laboratory; in this work, we suggest the use of Ustilaginomycetes as model organisms for studying fungi multicellularity.

## Figures and Tables

**Table 1:** Signal transduction pathways related to the multicellular growth of Ustilaginomycetes.

Pathway	Gene	*Ustilago maydis* ^a^	*Ustilago hordei* ^a,b^	*Ustilago bromivora* ^c^	*Sporisorium reilianum* ^a,b^	*Sporisorium graminicola* ^a^	*Testicularia cyperi* ^a,b,d^
**PKA**	*RAL2*	UMAG_00884	UHOR_01334	UBRO_01334	sr12177	EX895_006321	BCV70DRAFT_173605
	*GPA3*	UMAG_04474	UHOR_06981	UBRO_06981	sr15360	EX895_002426	CE102777_5455
	*BPP1*	UMAG_00703	UHOR_01085	UBRO_01086	sr11991	EX895_000934	CE92962_8479
	*PKA*	UMAG_06450	UHOR_09003	UBRO_09003	sr16888	EX895_004218	CE31316_43035
	*UAC1*	UMAG_05232	UHOR_03218	UBRO_03218	sr13141	EX895_004629	BCV70DRAFT_200755
	*UBC1*	UMAG_00525	UHOR_00805	UBRO_00805	sr10199.2	EX895_000753	BCV70DRAFT_164254
	*ADR1*	UMAG_04456	UHOR_06957	UBRO_06957	sr15343	EX895_002409	BCV70DRAFT_103006
	*SQL1*	UMAG_05501	UHOR_07888	UBRO_07888	sr16182	EX895_002861	BCV70DRAFT_212144
	*HGL1*	UMAG_11450	UHOR_00981	UBRO_00981	sr11921	EX895_000866	BCV70DRAFT_202631
	*CRK1*	UMAG_11410	UHOR_04041	UBRO_04041	sr10962	EX895_005166	BCV70DRAFT_157158
**MAPK**	*RAS2*	UMAG_01643	UHOR_01498	UBRO_02437	sr12711	EX895_004437	CE98444_23931
	*SQL2*	UMAG_10803	UHOR_02247	UBRO_02247	sr12585	EX895_004314	BCV70DRAFT_231562
	*SQL2*	UMAG_11476	UHOR_04466	UBRO_04466	sr13877	EX895_001846	BCV70DRAFT_223969
	*UBC2*	UMAG_05261	UHOR_08070	UBRO_08070	sr16309	EX895_003017	BCV70DRAFT_197656
	*MAPKKK*	UMAG_04258	UHOR_06391	UBRO_06391	sr15150	EX895_002006	BCV70DRAFT_154027
	*MAPKK*	UMAG_01514	UHOR_02245	UBRO_02245	sr12583	EX895_004312	BCV70DRAFT_211523
	*MAPKK*	UMAG_11453	UHOR_01494	UBRO_00992	sr11928	EX895_000873	CE125301_8671
	*MAPKK*	UMAG_00721	UHOR_01105	UBRO_01105	sr12009	EX895_000953	BCV70DRAFT_71012
	*MAPK*	UMAG_03305	UHOR_05144	UBRO_05144	sr14305	EX895_005556	CE107062_12366
	*MAPK*	UMAG_02331	UHOR_05144	UBRO_05144	sr14305	EX895_005556	BCV70DRAFT_107063
	*CRK1*	UMAG_11410	UHOR_04041	UBRO_04041	sr10962	EX895_005166	BCV70DRAFT_157158
**RIM**	*RIM101*	UMAG_10426	UHOR_06410	UBRO_06410	sr15166	EX895_002022	CE711_11906
	*RIM9*	UMAG_00581	UHOR_00899	UBRO_00899	sr11859	EX895_000793	CE73867_14878
	*RIM20*	UMAG_11510	UHOR_05885	UBRO_05885	sr14775	EX895_001317	BCV70DRAFT_202631
	*RIM13*	UMAG_02075	UHOR_03447	UBRO_03447	sr13297	EX895_003420	BCV70DRAFT_198765
	*RIM23*	UMAG_04392	UHOR_06863	UBRO_06863	sr15280	EX895_002350	BCV70DRAFT_161602
	*RHO4*	UMAG_10663	UHOR_01182	UBRO_05736	sr14670	EX895_001201	CE23374_4310

ID genes according to NCBI (^a^), ExPASy (^b^), e!EnsemblFungi (^c^), and JGI MycoCosm (^d^), and considering the genomic data published for *U. maydis* [66], *U. hordei* [67], *U. bromivora* [68], *S. reilianum* [69], *S. graminícola* [70], and *T. cyperi* [71]; the six Ustilaginomycetes with the best-annotated genomes. Genes identified based on their homology with *U. maydis*. The sequences analyzed were deposited in https://github.com/lucilaortiz/Multicellularity_associated_proteins.

**Table 2:** Genes involved in the polyamine synthesis pathway of Ustilaginomycetes.

Gene		*Ustilago* *maydis* ^a^	*Ustilago* *hordei* ^a,b^	*Ustilago* *bromivora* ^c^	*Sporisorium* *reilianum* ^a,b^	*Sporisorium* *graminicola* ^a^	*Testicularia* *cyperi* ^a,b,d^
Acetylornithine aminotransferase	*OAT*	UMAG_05671	UHOR_07330	UBRO_07330	sr15996	EX895_002715	BCV70DRAFT_201257
Ornithine decarboxylas	*ODC*	UMAG_01048	UHOR_01580	UBRO_01580	sr12348	EX895_006099	BCV70DRAFT_212462
Polyamine oxidase	*PAO*	UMAG_05850	UHOR_08347	UBRO_08347	sr16473	EX895_008244	BCV70DRAFT_173429
Chimeric spermidine synthase/saccharopine reductase	*SPE*	UMAG_05818	UHOR_08297	UBRO_08297	sr16440	EX895_003211	BCV70DRAFT_199161
S-adenosylmethionine decarboxylase	*SAMDC*	UMAG_10792	UHOR_01520	UBRO_01520	sr12300	EX895_006201	BCV70DRAFT_205658
Putative spermidine acetyltrasferase	*SSAT*	UMAG_00127	UHOR_00200	UBRO_00200	sr11469	EX895_000152	BCV70DRAFT_169192

ID genes according to NCBI (^a^), ExPASy (^b^), e!EnsemblFungi (^c^), and JGI MycoCosm (^d^), and considering the genomic data published for *U. maydis* [66], *U. hordei* [67], *U. bromivora* [68], *S. reilianum* [69], *S. graminícola* [70], and *T. cyperi* [71]; the six Ustilaginomycetes with the best-annotated genomes. Genes identified based on their homology with *U. maydis*. The sequences analyzed were deposited in https://github.com/lucilaortiz/Multicellularity_associated_proteins.

**Table 3:** Genes described as related with the multicellular growth of Ustilaginomycetes.

Gene	*Ustilago maydis* ^a^	*Ustilago hordei* ^a,b^	*Ustilago bromivora* ^c^	*Sporisorium reilianum* ^a,b^	*Sporisorium graminícola* ^a^	*Testicularia cyperi* ^a,b,d^
*GCN5*—Histone acetyltransferase	UMAG_05168	UHOR_03120	UBRO_03120	sr13072	EX895_004562	BCV70DRAFT_198404
Putative histone acetylase	UMAG_10190	UHOR_03120	UBRO_03120	sr13072	EX895_004562	BCV70DRAFT_198404
*CLR3*—Histone deacetylase	UMAG_02102	UHOR_03487	UBRO_03487	sr13325	EX895_004958	BCV70DRAFT_166948
*HOS2*—Histone deacetylase	UMAG_11828	UHOR_01015	UBRO_01015	sr11943	EX895_000889	BCV70DRAFT_70918
*TEA4*—SH3 domain protein	UMAG_01012	UHOR_01534	UBRO_01534	sr12319	EX895_006176	BCV70DRAFT_159133
*TEA1*—Kelch domain protein	UMAG_15019	UHOR_01124	UBRO_01124	sr12022	EX895_000966	BCV70DRAFT_69247
*REP1*—Repellent	UMAG_03924	UHOR_05948	UBRO_05948	sr14829	EX895_001361	BCV70DRAFT_80316
*REP4*—Repellent	UMAG_04517	UHOR_06499	UBRO_06499	sr15402	EX895_002086	BCV70DRAFT_202324
*HUM2*—Hydrophobin 2	UMAG_11562	UHOR_07158	UBRO_07158	sr15890	EX895_002555	BCV70DRAFT_207191
*HUM3*—Hydrophobin 3	UMAG_04433	UHOR_06926	UBRO_06926	sr15320	EX895_002383	BCV70DRAFT_189664
*RRM4*—RNA-binding protein	UMAG_03494	UHOR_05377	UBRO_05377	sr14484	EX895_006466	BCV70DRAFT_213009
Actin-binding protein	UMAG_05340	UHOR_08160	UBRO_08160	sr16324	EX895_003081	BCV70DRAFT_155405
Actin	UMAG_11232	UHOR_08813	UBRO_08813	sr11345	EX895_003701	BCV70DRAFT_201455
Actin-interactin gprotein; actin patch component	UMAG_05949	UHOR_08467	UBRO_08467	sr16579	EX895_005119	BCV70DRAFT_213703
Actin filament organization	UMAG_04613	UHOR_06638	UBRO_06638	sr15500	EX895_002183	BCV70DRAFT_202237
*LIS1*—Nuclear migration	UMAG_03164	UHOR_04938	UBRO_04938	sr14206	EX895_005820	BCV70DRAFT_200264
Actin regulating Ser/Thr kinase	UMAG_03081	UHOR_04824	UBRO_04824	sr14140	EX895_002483	-
Formin; actin nucleation	UMAG_12254	UHOR_06202	UBRO_06202	sr15020	EX895_001591	BCV70DRAFT_205747
Formin; actin nucleation. Functionally redundant with Bni1	UMAG_01141	UHOR_01723	UBRO_01723	sr12440	EX895_006065	BCV70DRAFT_12160
F-actin capping protein alpha subunit	UMAG_00423	UHOR_00664	UBRO_00664	sr11788	EX895_000652	BCV70DRAFT_196835
F-actin capping protein beta subunit	UMAG_11177	UHOR_08001	UBRO_08001	sr16266	EX895_002944	BCV70DRAFT_201290
Chaperonin role for actin and tubulin	UMAG_01279	UHOR_01918	UBRO_01918	sr10401	EX895_001741	BCV70DRAFT_197510
Chaperonin role for actin and tubulin	UMAG_06235	UHOR_08845	UBRO_08845	sr16767	EX895_003720	BCV70DRAFT_199599
Chaperonin role for actin and tubulin	UMAG_06067	UHOR_08612	UBRO_08612	sr16694	EX895_003550	BCV70DRAFT_202419
Chaperonin role for actin and tubulin	UMAG_02571	UHOR_04113	UBRO_04113	sr17132	EX895_005225	BCV70DRAFT_198632
Chaperonin role for actin and tubulin	UMAG_03959	UHOR_06000	UBRO_06000	sr14864	EX895_001395	BCV70DRAFT_69984
Chaperonin role for actin and tubulin	UMAG_02350	UHOR_03838	UBRO_03838	sr13553	EX895_004884	BCV70DRAFT_198957
Chaperonin role for actin and tubulin	UMAG_00565	UHOR_00911	UBRO_00911	sr11843	EX895_000809	BCV70DRAFT_73687
Chaperonin role for actin and tubulin	UMAG_04401	UHOR_06879	UBRO_06879	sr15289	EX895_002355	BCV70DRAFT_200184
Actin binding and severing protein	UMAG_04314	UHOR_06753	UBRO_06753	sr15202	EX895_002269	BCV70DRAFT_211263
Cortical actin cytoskeleton component	UMAG_04417	UHOR_06902	UBRO_06902	sr15303	EX895_002369	BCV70DRAFT_200171
Kinesin—Related to UMAG_ Kin14	UMAG_11986	UHOR_06484	UBRO_06484	sr15392	EX895_002076	BCV70DRAFT_202332
Kinesin—Functionally redundant with Cin8	UMAG_10678	UHOR_07574	UBRO_07574	sr15603	EX895_000239	BCV70DRAFT_203263
Kinesin—Related motor protein (UMAG_ Kin7a and Kin7b)	UMAG_00896	UHOR_01350	UBRO_01350	sr12193	EX895_006269	BCV70DRAFT_199338
Kinesin—related motor protein / Related to UMAG_ Kin8	UMAG_01560	UHOR_02319	UBRO_02319	sr12632	EX895_004356	BCV70DRAFT_199992
Interacts with Myo2—related to motor domain of kinesins	UMAG_04218	UHOR_06328	UBRO_06328	sr15103	EX895_001669	-
Type V myosin	UMAG_04555	UHOR_06551	UBRO_06551	sr15438	EX895_002121	BCV70DRAFT_40829
*MEP1*—Low affinity ammonium transporter	UMAG_04523	UHOR_06506	UBRO_06506	sr15408	EX895_002092	BCV70DRAFT_208136
*MEP2*—High affinity ammonium transporter	UMAG_05889	UHOR_08388	UBRO_08388	sr16507	EX895_003276	BCV70DRAFT_199125
Putative arginase—Homology with *G6606* of *U. esculenta*	UMAG_04939	UHOR_07065	UBRO_07065	sr15821	EX895_005725	BCV70DRAFT_178133
Homology with *SSAGC1* (SPSC_00276) of *S. scitamineum*)	UMAG_11677	UHOR_02215	UBRO_02215	sr12574	EX895_004280	BCV70DRAFT_160776
*CDC24*—Cell division control protein	UMAG_02422	UHOR_03963	UBRO_03963	sr10836	EX895_004832	BCV70DRAFT_192816
*CLA4*—Serine/threonine-protein kinase	UMAG_10145	UHOR_03893	UBRO_03893	sr10814.2	EX895_004846	BCV70DRAFT_158032
*RHO1*—Ras-like GTP-binding protein	UMAG_01032	UHOR_01559	UBRO_01559	sr12336	EX895_006088	BCV70DRAFT_166498
*RHO1*—Ras-like GTP-binding protein	UMAG_05734	UHOR_12739	UBRO_07439	sr16067	EX895_002745	CE53947_104590
FUZ1—MYND domain protein	UMAG_02587	UHOR_04136	UBRO_04136	sr13628	EX895_005246	BCV70DRAFT_63386

Genes mostly analyzed by its deletion. ID genes according to NCBI (^a^), ExPASy (^b^), e!EnsemblFungi (^c^), and JGI MycoCosm (^d^), and considering the genomic data published for *U. maydis* [66], *U. hordei* [67], *U. bromivora* [68], *S. reilianum* [69], *S. graminícola* [70], and *T. cyperi* [71]; the six Ustilaginomycetes with the best-annotated genomes. Genes identified based on their homology with *U. maydis*. The analyzed sequences were deposited in https://github.com/lucilaortiz/Multicellularity_associated_proteins.

**Table 4:** Genes involved in synthesis and degradation of the cell wall, and putatively involved in the Ustilaginomycetes multicellular growth.

Cell wall	Gene	*Ustilago maydis* ^a^	*Ustilago* *hordei* ^a,b^	*Ustilago* *bromivora* ^c^	*Sporisorium* *reilianum* ^a,b^	*Sporisorium* *graminicola* ^a^	*Testicularia* *cyperi* ^a,b,d^
Synthesis	*1,3-β-glucan synthase*	UMAG_01639	UHOR_02430	UBRO_02430	sr12707	EX895_004433	BCV70DRAFT_98536
	*CHS1*	UMAG_10718	UHOR_07282	UBRO_07282	sr16010	EX895_002642	BCV70DRAFT_198128
	*CHS2*	UMAG_04290	UHOR_06435	UBRO_06435	sr15181	EX895_002037	-
	*CHS3*	UMAG_10120	UHOR_00740	UBRO_00740	sr10158	EX895_000703	BCV70DRAFT_196781
	*CHS4*	UMAG_10117	UHOR_00723	UBRO_00723	sr10139	EX895_000691	CE2605_17934
	*CHS5*	UMAG_10277	UHOR_04112	UBRO_04112	sr11048.2	EX895_005224	BCV70DRAFT_156817
	*CHS6*	UMAG_10367	UHOR_04780	UBRO_04780	sr14109	EX895_002515	BCV70DRAFT_22903
	*CHS7*	UMAG_05480	UHOR_07854	UBRO_07854	sr16158	EX895_002841	BCV70DRAFT_162870
	*CHS8*	UMAG_03204	UHOR_04988	UBRO_04988	sr11106	EX895_005864	BCV70DRAFT_105640
	*CDA1* ^d^	UMAG_11922	UHOR_04296	UBRO_04296	sr13741	EX895_005345	BCV70DRAFT_210413
	*CDA2* ^d^	UMAG_00126	UHOR_00199	UBRO_00199	sr11468	EX895_000151	BCV70DRAFT_197174
	*CDA3* ^d^	UMAG_00638	-	-	sr11918	EX895_000863	-
	*CDA4* ^d^	UMAG_01143	UHOR_01725	UBRO_01725	sr12442	EX895_006063	-
	*CDA5* ^d^	UMAG_01788	UHOR_02660	UBRO_02660	sr12866	EX895_003869	BCV70DRAFT_211320
	*CDA6* ^d^	UMAG_02019	UHOR_03000	UBRO_03000	sr12981	EX895_004113	BCV70DRAFT_34433
	*CDA7* ^d^	UMAG_02381	UHOR_03898	UBRO_03898	sr13587	EX895_004867	BCV70DRAFT_163992
	*CDA8* ^d^	UMAG_05792	UHOR_04296	UBRO_04296	sr16123	EX895_002800	BCV70DRAFT_210413
Degradation	*CTS1*	UMAG_10419	UHOR_06394	UBRO_06394	sr15153	EX895_002009	BCV70DRAFT_196730
	*CTS2*	UMAG_02758	UHOR_04393	UBRO_04393	sr13813	EX895_005415	CE60997_75476
	*CTS3*	UMAG_06190	UHOR_08772	UBRO_06394	sr11305	EX895_003673	BCV70DRAFT_178298
	*CTS4*	UMAG_00695	UHOR_01069	UBRO_01069	sr11983	EX895_000921	BCV70DRAFT_213542
	*CTS5*	UMAG_05290	UHOR_08119	UBRO_08119	sr10043	EX895_003044	BCV70DRAFT_197812
	*Glucanase 1*	UMAG_04368	-	UBRO_06828	sr15253	-	-
	*Glucanase 2*	UMAG_05036	UHOR_07211	UBRO_07211	sr15917	-	CE121843_61034
	*Glucanase 3*	UMAG_00876	UHOR_00370	UBRO_01319	sr12165	EX895_006333	CE84129_149800
	*Glucanase 4*	UMAG_01898	-	UBRO_02820	sr10624	EX895_003984	-
	*Glucanase 5*	UMAG_05223	UHOR_03204	UBRO_03204	sr13131	EX895_004619	BCV70DRAFT_227049
	*Glucanase 6*	UMAG_02134	-	UBRO_03533	sr13360	EX895_004991	-
	*Glucanase 7*	UMAG_04357	UHOR_06809	UBRO_06809	sr15243	EX895_002306	BCV70DRAFT_189826
	*Glucanase 8*	UMAG_05550	-	UBRO_06823	sr15250	EX895_002316	BCV70DRAFT_200231
	*Glucanase 9*	UMAG_10211	-	UBRO_08206	sr16359	EX895_003114	-

ID genes according to NCBI (^a^), ExPASy (^b^), e!EnsemblFungi (^c^), and JGI MycoCosm (^d^), and considering the genomic data published for *U. maydis* [66], *U. hordei* [67], *U. bromivora* [68], *S. reilianum* [69], *S. graminícola* [70], and *T. cyperi* [71]; the six Ustilaginomycetes with the best-annotated genomes. Genes identified based on their homology with *U. maydis*. *CHS*, Chitinsynthase; *CDA*, Chitindeacetylase; and *CTS*, Chitinase. The sequences analyzed were deposited in https://github.com/lucilaortiz/Multicellularity_associated_proteins.
